# Monocytes co-cultured with reconstructed keloid and normal skin models skew towards M2 macrophage phenotype

**DOI:** 10.1007/s00403-019-01942-9

**Published:** 2019-06-11

**Authors:** Grace C. Limandjaja, Taco Waaijman, Sanne Roffel, Frank B. Niessen, Susan Gibbs

**Affiliations:** 1 Department of Molecular Cell Biology and Immunology, O|2 Lab Building Room 11E05, Amsterdam University Medical Centre (UMC), Vrije Universiteit Amsterdam, Amsterdam Movement Sciences, De Boelelaan, 1108 Amsterdam, The Netherlands; 2 Department of Plastic Surgery, Amsterdam UMC, Vrije Universiteit Amsterdam, Amsterdam, The Netherlands; 3 Department of Oral Cell Biology, Academic Centre for Dentistry Amsterdam (ACTA), University of Amsterdam and Vrije Universiteit Amsterdam, Amsterdam, The Netherlands

**Keywords:** In vitro model, Co-culture, Organotypic, Keloid, Immune cells, Monocytes, PBMC, M2 macrophages

## Abstract

Several abnormalities have been reported in the peripheral blood mononuclear cells of keloid-forming patients and particularly in the monocyte cell fraction. The goal of this in vitro study was to determine whether monocytes from keloid-prone patients contribute to the keloid phenotype in early developing keloids, and whether monocyte differentiation is affected by the keloid microenvironment. Therefore, keloid-derived keratinocytes and fibroblasts were used to reconstruct a full thickness, human, in vitro keloid scar model. The reconstructed keloid was co-cultured with monocytes from keloid-forming patients and compared to reconstructed normal skin co-cultured with monocytes from non-keloid-formers. The reconstructed keloid showed increased contraction, dermal thickness (trend) and α-SMA+ staining, but co-culture with monocytes did not further enhance the keloid phenotype. After 2-week culture, all monocytes switched from a CD11c^high^/CD14^high^/CD68^low^ to a CD11c^high^/CD14^low^/CD68^high^ phenotype. However, only monocytes co-cultured with either reconstructed keloid scar or normal skin models skewed towards the more fibrotic M2-macrophage phenotype. There was negligible fibroblast and fibrocyte differentiation in mono- and co-cultured monocytes. These results indicate that monocytes differentiate into M2 macrophages when in the vicinity of early regenerating and repairing tissue, independent of whether the individual is prone to normal or keloid scar formation.

**Electronic supplementary material:**

The online version of this article (10.1007/s00403-019-01942-9) contains supplementary material, which is available to authorized users.

## Introduction

Keloid scar formation remains a poorly understood complication of abnormal wound healing [27, 30, 32], and further research into its pathogenesis is severely hampered by the lack of a suitable in vivo-like scar model. As keloids occur exclusively in humans [22, 36], there is a need for relevant human in vitro models. We have previously utilized our skin tissue engineering expertise to construct a basic keloid scar model, consisting of a reconstructed epidermis on a fibroblast-populated dermal matrix with keratinocytes and fibroblasts derived from excised keloid scars [17]. However, the differences we observed between our normal skin and keloid scar models were not of the order of magnitude one would expect from such a decidedly abnormal type of scar such as a keloid. This therefore suggests that the intrinsic abnormalities associated with keloid keratinocytes and keloid fibroblasts are not the only causative agents involved in keloid formation. Given the fact that normal wound healing processes involve many different types of cells and a myriad of secreted wound healing factors, it is very likely that keloid scar formation would require more than abnormal keratinocytes and fibroblasts.

Most research conducted on the underlying pathogenesis of keloid scarring has focused solely on the keloid fibroblast and its excessive extracellular matrix production [3, 16]. Although the immune system is known to play an essential role in normal wound healing processes [4, 19], not much effort has been directed towards elucidating the possible role of the immune system in keloid formation or the presence of intrinsic abnormalities in the immune system of keloid patients [22]. However, recent studies seem to support the role of immune cells in keloid formation. For example, immunohistochemical studies found that keloids were characterized by an increase in the number of monocytes [15], macrophages, T-lymphocytes (with increased CD4+:CD8+ ratio) and mast cells compared to normal skin [1, 3, 10, 11, 31]. Variable results have been reported varying from relatively few [3, 31] to increased levels of B-lymphocytes [1] in keloid scars compared to normal skin. There is also evidence for the presence of intrinsic immunological abnormalities in patients with a history of keloid formation after trauma. McCauley and colleagues [22] compared the production of various cytokines by peripheral blood mononuclear cells (PBMCs) derived from peripheral venous blood of dark-skinned keloid patients to that of dark-skinned healthy controls. PBMCs from keloid patients showed increased production of IL-6, TNF-α and IFN-β, a decreased production of IFN-α, IFN-γ and TNF-β, but no difference in IL-1 or IL-2 production compared to healthy controls. Interestingly, PBMCs can also differentiate into a particular leukocyte subtype with fibroblast-like characteristics called fibrocytes [5, 21] and there is evidence that these fibrocytes behave abnormally in keloid scars as well. Compared to control subjects, Naylor et al. found that keloid PBMCs showed an almost a 20-fold increase in fibrocyte differentiation when cultured in vitro in serum-free medium [23]. Additionally, dermal cells isolated from keloid scar tissue comprised higher number of fibrocytes compared to normal scar tissue [9].

Based on the aforementioned results, it would be expected that the addition of these intrinsically abnormal immune cells from keloid-forming patients to the basic keloid scar model may help induce a more extreme keloid scar phenotype. Although various cell types fall under the denominator ‘PBMC’, the CD14+ monocyte subset has been reported to be capable of differentiating into the aforementioned fibrocytes [7, 37]. For this reason, CD14+ monocytes isolated from PBMCs from keloid formers were used for co-culture with our reconstructed keloid scar (Kscar) model [17] to determine whether they could enhance the in vitro keloid phenotype as previously established (increased contraction, dermal thickness and α-SMA immunoreactivity; reduced dermal collagen-type IV α2, hyaluronan synthase 1 and matrix metalloproteinase 3 gene expression; reduced HGF secretion). Additionally, we determined whether the reconstructed keloid could affect the phenotype of the co-cultured monocytes. Monocytes from normal scar-forming subjects co-cultured with reconstructed normal skin (Nskin) models served as a control group. Since the monocytes were co-cultured with reconstructed keloid scar and normal skin models as these skin tissues were developing over the 5-week culture period, these models can be considered to represent the early phase of tissue remodelling.

Immunophenotyping was performed before and after (co)-culture to assess potential monocyte differentiation into macrophages in general (CD68+), classically activated M1 macrophages (CD40+/CD68+) thought to be important in the early phases of wound healing [34], and alternatively activated M2 macrophages (CD206+/CD68+) associated with tissue repair [8, 34] and fibrosis [24, 25], as well as fibrocytes with both classic fibrocyte markers (CD34+/LSP-1+/collagen 1+ and CD45+/LSP-1+/collagen 1+) [5, 21, 23] and new fibrocyte markers (combination of intra- and extracellular CD45RO+/25F9+/MRP8/14+/PM2K−) [9, 26]. Additionally, the presence of classic monocyte markers (CD14 and CD11c) and dendritic cell markers (CD1a) was evaluated before and after culturing.

## Materials and methods

### Tissue

Normal skin was obtained from patients undergoing body contouring surgery to remove excess skin, and the discarded skin was collected anonymously. Keloid scars were obtained from patients undergoing keloid removal via excision, and selection was performed by an experienced scar expert (plastic surgeon, author FBN). The discarded keloid tissue was coded to enable the collection of additional relevant information (e.g. previous treatment, age of keloid). Both normal skin and keloid tissue were collected after obtaining informed consent, and collection procedures were in compliance with the ‘Code for Proper Secondary Use of Human tissue’ as formulated by the Dutch Federation of Medical Scientific Organization (www.fmwv.nl) and with the approval of the local medical research ethics committee (MREC) of the Amsterdam UMC.

### Monocytes

For isolation of monocytes from peripheral blood, all patients included were at least 18 years old and capable of giving written informed consent. Reconstructed normal skin models were co-cultured with normal monocytes from healthy volunteers not prone to keloid scarring. To establish subjects that were not prone to keloid formation, the formation of a normal scar of at least 5 cm in length and at least 6 months after inciting trauma (surgery of the abdomen or thorax) was considered sufficient evidence to qualify as a non-keloid-forming control subject. Reconstructed keloid scar models were co-cultured with monocytes derived from keloid-forming patients. Patients were considered keloid prone when they presented with a history of keloid formation, defined as the presence of at least one major keloid (> 0.5 cm), with the keloid diagnosed as such by an experienced scar expert (plastic surgeon, author FBN). Exclusion criteria for all monocyte donors were: systemic illness and chronic use of systemic medication (e.g. corticosteroids, anti-inflammatory drugs such as aspirin). Donor characteristics of the tissue samples and monocytes used are summarized in Table 1 and, with the exception of one keloid donor (no. 11), tissues and monocytes were not donor matched. All procedures outlined in this study were approved by the Amsterdam UMC medical ethics board (MREC).

**Table 1:** Tissue and monocyte donor characteristics

Donor	Tissue	Location	Aetiology	Age	Previous treatment	Skin colour	Pt age	Gender
1 2	Nskin N mono	Abdomen NA	NA NA	NA NA	NA NA	Unknown White	44 yrs 53 yrs	Female Female
3 4	Nskin N mono	Abdomen NA	NA NA	NA NA	NA NA	Unknown White	41 yrs 50 yrs	Female Female
5 6	Nskin N mono	Unknown NA	NA NA	NA NA	NA NA	White Unknown	42 yrs 50 yrs	Female Female
7 8	Kscar Ks mono	Abdomen NA	Surgery NA	2 yrs NA	None NA	Dark brown Dark brown	40 yrs 55 yrs	Female Female
9 10	Kscar Ks mono	Earlobe NA	Piercing NA	4 yrs NA	None NA	Dark brown Dark brown	20 yrs 20 yrs	Male Female
11^a^ 12^a^	Kscar Ks mono	Sternum NA	Surgery NA	10 yrs NA	Corticosteroids NA	Dark brown Dark brown	26 yrs 26 yrs	Female Female

Overview of the characteristics of the tissue samples used for the construction of the SE. All the skin models were constructed using donor-matched keratinocytes and fibroblasts; note that each tissue donor is followed by the co-cultured monocyte donor below so that each table row represents the combination of donors used per co-culture

*NA* not applicable, *Donor age* in years (yrs), *Nskin* normal skin, *Kscar* keloid scar, *N mono* normal monocytes, *Ks mono* keloid monocytes

^a^The only SE and mono from the same donor

### Cell culture

For keloids, subcutaneous fat and any other soft tissue were removed until the typical firm and rubbery keloid consistency was reached. They were then were dissected into smaller fragments for further processing. Keratinocytes and fibroblasts were isolated from normal skin and keloids as described previously [17, 35] and cultured in a 37 °C, 7.5% CO_2_ atmosphere with KC-I medium and in a 37 °C, 5% CO_2_ atmosphere with fibroblast medium, respectively. The medium was changed twice a week; see supplementary Table 1 for contents of culture media used.

**Fig. 1:**
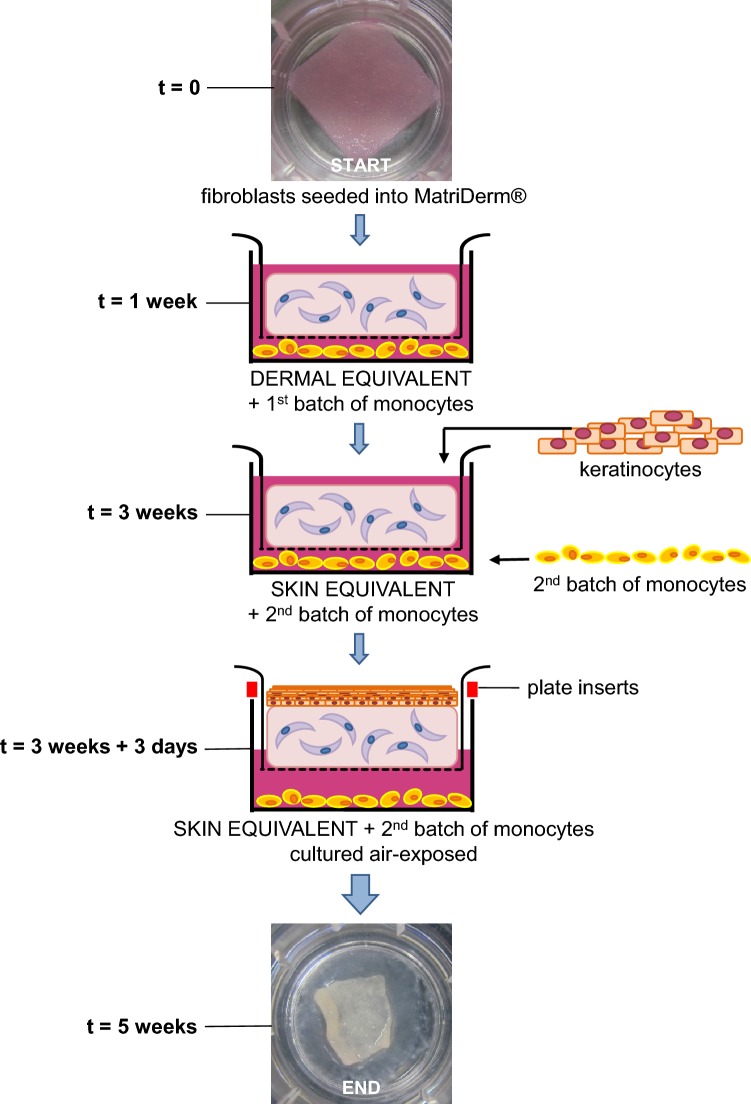

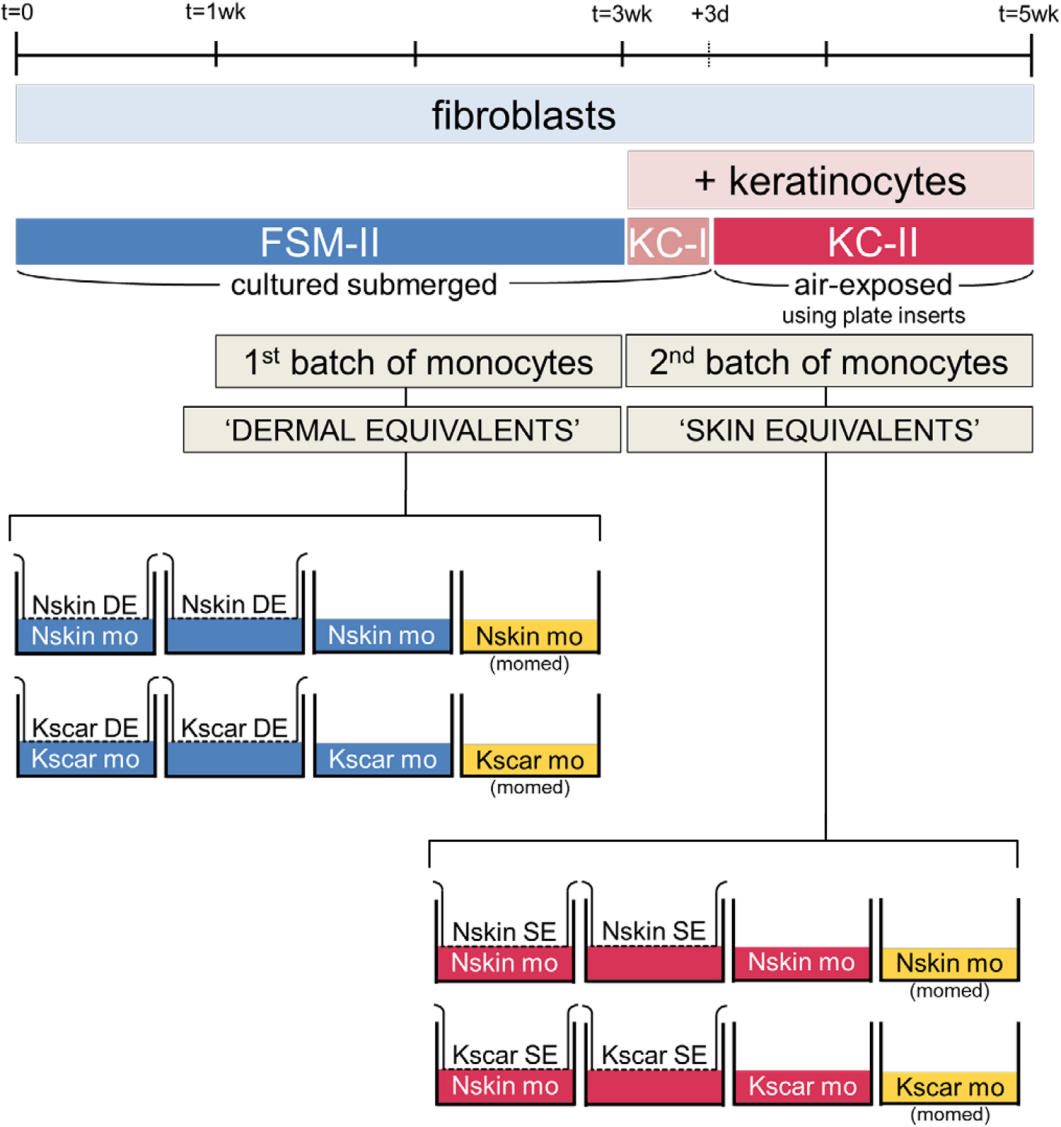
Construction of skin models. **a** Skin models are constructed by first seeding fibroblasts into MatriDerm^®^ (first picture: macroscopic view). After 3 weeks, keratinocytes are added and the skin models are then cultured air-exposed for an additional 2 weeks. The first batch of monocytes was added during week 1–3 (only fibroblasts present, DE stage) and the second fresh batch of monocytes during week 3–5 (fibroblasts and keratinocytes present, SE stage). The last macroscopic image shows the same skin model as shown above, at 5 weeks after initiating culture. The diameter of the transwell insert is 24 mm. **b** Overview of the culturing process during the entire 5-week timeline as explained in ‘Materials and methods’. The first batch was co-cultured with DE and second monocyte batch was co-cultured with SE. The experimental conditions for both stages are depicted in the final row. From left to right, Nskin/Kscar skin models were co-cultured with their respective monocytes for 2 weeks (SE/DE + mo) and compared to the three control conditions: skin models only (SE/DE), monocytes only cultured in either the same medium as the skin models (mo) or in their own medium (mo, mo-medium). *wk* week, *d* days, *DE* dermal equivalents, *SE* skin equivalents, *mo* monocytes, *Nskin* normal skin, *Kscar* keloid scar, *mo*-*medium* monocyte medium, FSM-II, KC-I and KC-II: various types of media used, see supplementary Table 1

Peripheral blood from healthy volunteers not prone to keloid formation (N mono) and keloid-forming patients (Ks mono) was collected in 10 ml lithium–heparin plasma tubes (BD Biosciences, Bedford, MA, USA) for the isolation of peripheral blood mononuclear cells (PBMC). PBMCs were then isolated using a Ficoll (Lymphoprep™, Axis-Shield, Oslo, Norway) density gradient and then cryopreserved in liquid nitrogen until further use. On the day of co-culture with the skin models, monocytes were isolated from thawed PBMCs using human anti-CD14 MicroBeads for MACS^®^ separation (Miltenyi Biotec, Leiden, The Netherlands) according to the manufacturer’s protocol. Two batches of monocytes from the same donor were used for co-culture: monocytes were first added between culture period *t* = 1–3 weeks, then removed and replaced with a fresh batch of monocytes between culture period *t* = 3–5 weeks as described below (see Fig. 1a, b).

### Construction of skin models and co-culture with monocytes

Skin models were constructed (see Fig. 1a, b) in duplicate from keratinocytes (P2) and fibroblasts (P2–3) derived from *n* = 3 normal skin (Nskin) and *n* = 3 keloid (Kscar), essentially as previously described [17, 33]. In brief, 4 × 10^5^ fibroblasts were seeded onto 2.2 × 2.2 cm squares of MatriDerm^®^ (Dr. Suwelack Skin & Health Care, Billerbeck, Germany) with FSM-I and cultured submerged in FSM-II medium for 3 weeks in 0.4 µm pore size transwells (Costar Corning Inc., New York, NY, USA) in a 37 °C, 5% CO_2_ atmosphere. After 1 week, the first batch of 1 × 10^6^ monocytes were co-cultured in the wells underlying the skin models for 2 weeks. These fibroblast-populated MatriDerm^®^ skin models are referred to as dermal equivalents (DE) from here on. Keratinocytes (P2) were then seeded on top of the DE after an additional 2 weeks and a fresh second batch of 1 × 10^6^ monocytes (same donor as previously) were placed in the underlying wells. The skin equivalents (SE) and monocytes were cultured submerged in KC-I medium for 3–4 days, prior to culturing at an air–liquid interface in KC-II medium for an additional 10 days in a 37 °C, 7.5% CO_2_ atmosphere. For air-exposed culturing, customized polycarbonate inserts were used (supplemental Fig. 1) which raised the transwells on the six-well plate. This allowed for the addition of more medium in the underlying wells (without inserts: space for 2 ml in the underlying well; with inserts: 10 ml). The medium was changed twice a week; see supplemental Table 1 for contents of culture media used. As an additional control, monocytes were cultured without the SE in either (i) the same culture medium as the SE or (ii) in monocyte medium to evaluate the effects of SE culture medium on monocyte skewing. See Fig. 1 for an overview and timeline of the experimental setup.

### Immunophenotyping using flow cytometry

Monocyte phenotyping was performed by flow cytometry (FACS) both before co-culture and after each 2-week co-culture period with the skin models. The cells were stained with antibodies listed in supplemental Table 2. Both floating and adherent cells were included for phenotyping. Lidocaine hydrochloride 4 mg/ml (Sigma-Aldrich, St. Louis, MO, USA) and cell scrapers were used to detach the adherent cells from the wells. Cells were washed and resuspended in FACS buffer (PBS supplemented with 0.1% BSA and 0.1% sodium azide). FcR blocking reagent (Miltenyi Biotec, Leiden, The Netherlands) was added prior to incubation with the extracellular antibodies for the target markers and their corresponding isotypes for 30 min. at 4º. The majority of stainings were for extracellular markers, but some markers were inherently intracellular (LSP-1, collagen I, fibronectin, α-SMA), and some could be detected both intra- and extracellularly (MRP8/14, PM2K, CD45RO, 25F9). For intracellular staining, the BD Cytofix/Cytoperm^™^ Fixation/Permeabilization Kit (BD Biosciences) was used and permeabilization was performed according to the manufacturer’s protocol. Results were analysed using FACSCalibur and Cellquest-Pro FACS analysis software (BD Immunocytometry systems, Mountain view, CA, USA).

### Analysis of reconstructed skin models

As described previously [17], reconstructed normal skin and keloid scar models were evaluated with regard to (i) contraction: reduction in the surface area of the SE at the end of the culture period; (ii) epidermal thickness: number of viable keratinocyte cell layers; and (iii) dermal thickness: measured in µm in H&E-stained tissue sections; immunohistochemical staining to assess the presence of fibroblasts (vimentin) and myofibroblasts (α-SMA); (iv) secretion of IL-6, CXCL8, CCL2, CCL27, HGF and VEGF in the culture supernatants (2 ml KC-II medium without hydrocortisone or 2 ml of monocyte medium was collected over 24 h at the end of the culture period) by way of enzyme-linked immunosorbent assay (ELISA); (v) MTT assay: to quantify cell proliferation and viability of the SE. The following scoring method was used to evaluate immunohistochemical staining: (−) absence of staining; (+/− −) one or two positive cells; (+/−) minimal positive staining, predominantly negative staining; (+) normal staining pattern; (++) increased number of positively stained cells; (+++) strongly increased number of positively stained cells.

### Data analysis

Experiments were performed in duplicate with *n* = 3 different donors unless stated otherwise in the figure legends. For flow cytometry analysis, duplicate cultures were combined to ensure sufficient cell numbers for analysis. Immunohistochemical staining was also performed on only one of duplicate skin models. All results in graphs and tables were expressed as the mean ± standard error of the mean (SEM). Normality testing (Shapiro–Wilk test) was performed on the residuals (errors); the Kruskal–Wallis test with post hoc Dunn’s multiple comparisons test was applied if the residuals did not pass the normality test, while an ordinary one-way ANOVA with post hoc Tukey’s multiple comparisons test was employed if the residuals passed the normality test. Figure legends detail which test was used for the data displayed. For general analysis of the FACS data, post hoc comparisons were limited to ten in total to prevent further loss of power due to the high number of experimental groups (8 in total). Comparison pairs were selected to compare Kscar with Nskin for each experimental group (mo in momed, mo, mo + SE] and to compare the (co-) cultured monocytes (mo + SE) to those in the start population (mo START). For statistical analysis of the four main groups of interest (Nskin/Kscar monocytes mono-cultured in skin model medium, Nskin/Kscar monocytes co-cultured with skin models), testing was performed as initially stated above. Differences were considered significant if *p* < 0.05 (*), *p* < 0.01 (**), *p* < 0.001 (***) or *p* < 0.0001 (****). GraphPad Prism 6 software (GraphPad Software Inc., San Diego, CA, USA) was used to construct all graphs and tables and to perform statistical analysis. A trend was defined as an obvious pattern showing a difference between two groups which was not statistically significant (*p* < 0.08).

## Results

### Co-culture with monocytes did not affect normal skin or keloid phenotype

**Fig. 2:**
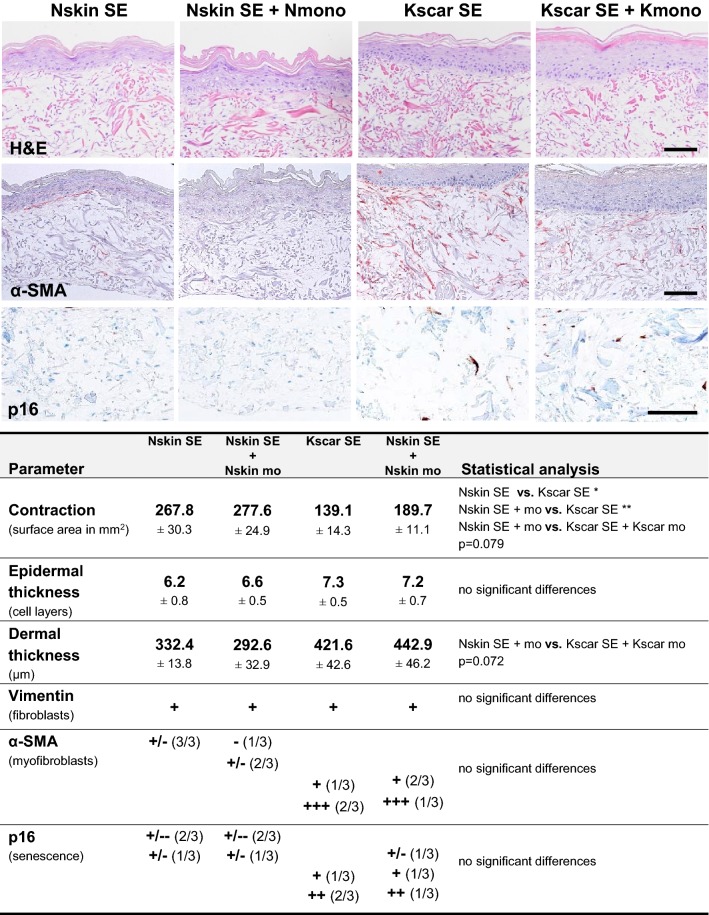
Effect of monocyte co-culture on the keloid scar phenotype. Images show immunohistochemical staining of normal skin equivalents cultured alone (Nskin SE) or with monocytes (Nskin SE + mo), and keloid skin equivalents cultured alone (Kscar SE) or with monocytes (Kscar SE + mo). The table summarizes the results for *n *= 3 normal skin (Nskin) and *n *= 3 keloid scars (Kscar), with or without monocytes (mo): contraction was measured as a reduction in the end surface area after a 5-week culture; epidermal thickness was measured as the number of viable epidermal cell layers in the SE; dermal thickness was measured in μm; vimentin staining indicated the presence fibroblasts within the MatriDerm^®^; α-SMA stained myofibroblasts; p16 was used as a senescence marker. Scoring legend for immunohistochemical staining: ± minimal expression, +: normal expression, ++ increased expression, +++ strongly increased expression, − absent. Results in the table were shown as mean ± SEM; vs.: versus (compared to). An ordinary one-way ANOVA with post hoc Tukey’s multiple comparisons test was performed with *p < 0.05, **p < 0.01. Bar = 100 µm

Previously, we reported increased contraction, dermal thickness (trend), increased expression of senescence marker *p*16 and α-SMA + myofibroblasts, but no apparent increase in epidermal thickness for keloid scar models when compared to normal skin models (manuscript in submission) [17]. In this study, H&E staining showed that the constructed normal skin and keloid scar models (co-cultured with or without monocytes) possessed a fully differentiated epidermis on top of a vimentin-positive, fibroblast-populated dermis (Fig. 2, supplemental Fig. 2). However, co-culture with monocytes did not affect contraction, dermal thickness, α-SMA expression, p16 expression or epidermal thickness in either normal skin or keloid scar (Fig. 2, supplemental Fig. 2).

**Fig. 3:**
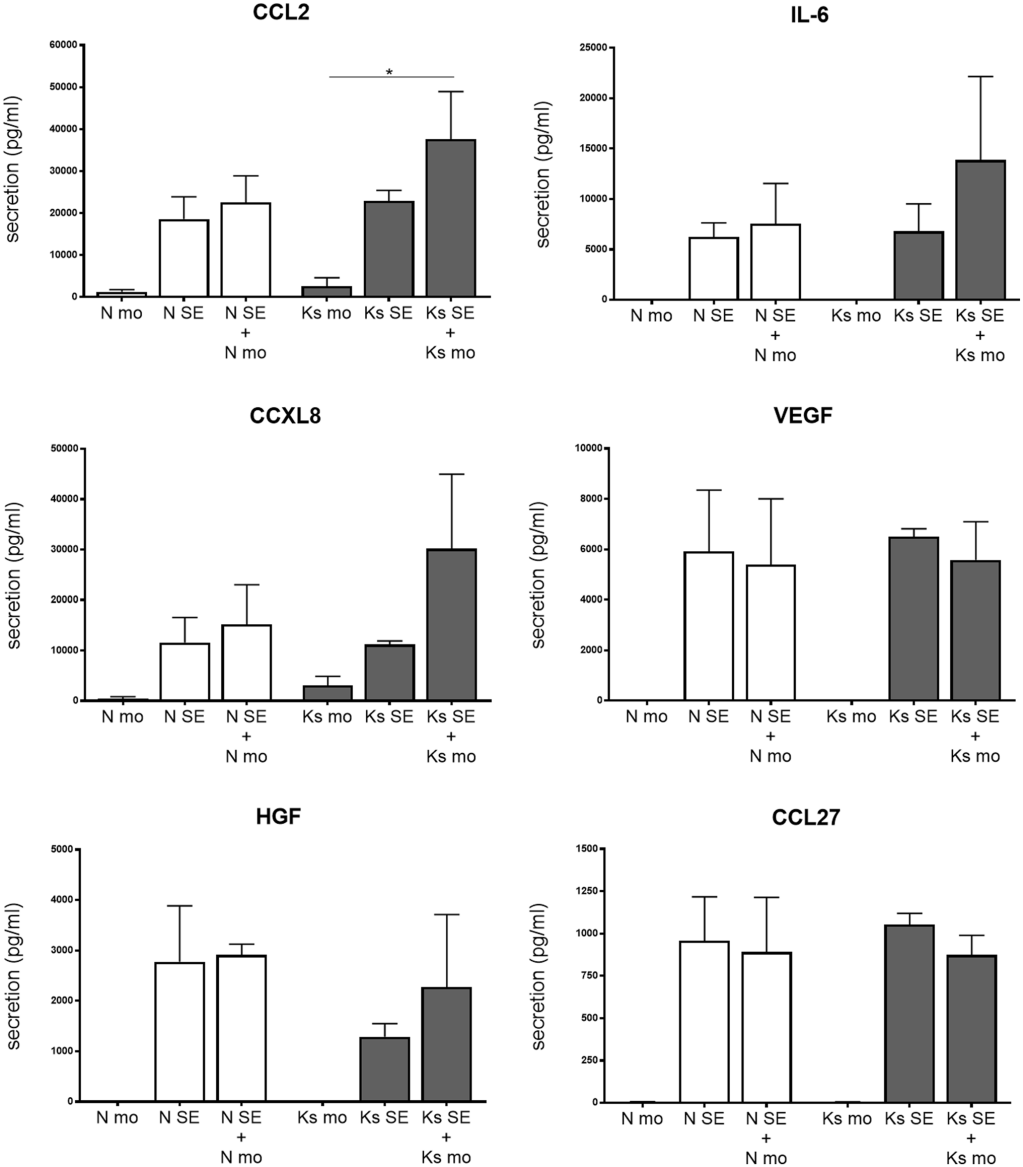
Differential secretion of wound healing mediators. Secretion of HGF, CCL27, IL-6, CXCL8, CCL2 and VEGF was determined in *n *= 3 normal skin (Nskin) and *n *= 3 keloid scars (Kscar) skin equivalents, with or without monocytes (mono). The Kruskal–Wallis test with post hoc Dunn’s multiple comparisons test (HGF, CCL27, IL-6, CXCL8, VEGF) or an ordinary one-way ANOVA with post hoc Tukey’s multiple comparisons test (CCL2) was employed. Graphs show mean ± SEM, with *p < 0.05. *SE* skin equivalents, *mo* monocytes, *N* normal skin, *Ks* keloid scar

We also examined secretion of a previously established wound healing mediator panel (CCL2, IL-6, CXCL8, VEGF, HGF and CCL27), which showed reduced HGF secretion in the reconstructed keloid model compared to the normal skin model [17]. It is readily apparent that all the cytokines and chemokines studied are virtually only secreted by the normal skin or keloid models, while monocytes cultured in isolation secrete no or negligible amounts of these wound healing factors (Fig. 3). There was no difference in the secretion levels of CCL2, IL-6, CXCL8, VEGF and CCL27 between the normal skin and keloid models in conformity with previous results, but HGF secretion was now not significantly decreased in the keloid model either. Also, there were no differences in secretion of wound healing factors between the monoculture of monocytes in their own monocyte medium or the skin model medium (data not shown). Most importantly, there was no difference between the secretion levels of the reconstructed skin or keloid co-cultured with their respective monocytes and the reconstructed models cultured without monocytes.

### CD11c^high^/CD14^high^/CD68^low^ monocytes differentiate into CD11c^high^/CD14^low^/CD68^high^ macrophage-like cells upon culture

**Fig. 4:**
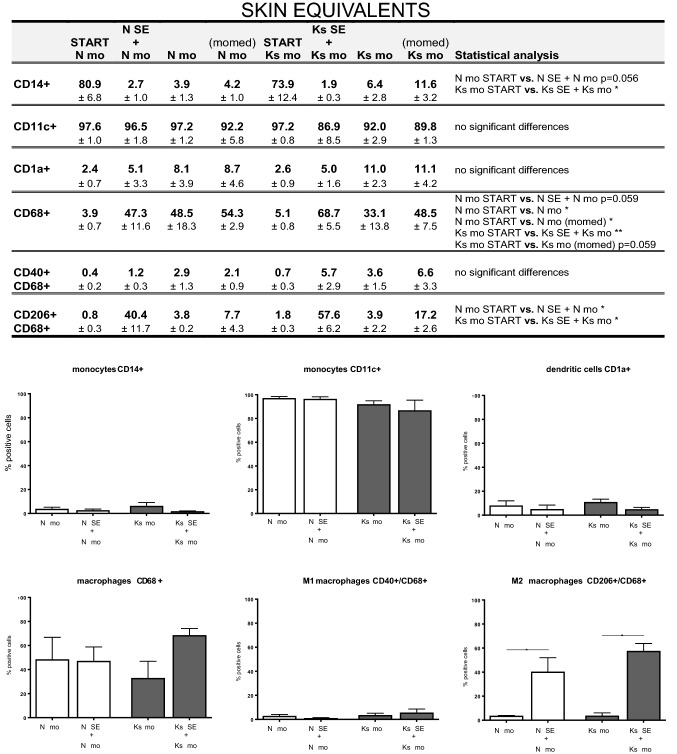
Immunophenotyping of monocytes (co-) cultured with full skin equivalents: monocyte, dendritic cell and macrophage marker expression. Monocytes, both mono- and co-cultured with skin equivalents, were analysed for monocyte (CD14+, CD11c+), dendritic cell (CD1a+), macrophage (CD68+), M1 macrophage (CD40+) and M2 macrophage (CD206+) marker expression via FACS analysis. The table summarizes the results of each experimental group by listing the mean % positive staining with SEM with *n *= 3 for all four normal skin conditions and *n *= 3 for all four keloid scar experimental groups; vs.: versus (compared to). Statistically significant results of an ordinary one-way ANOVA or Kruskal–Wallist test with post hoc testing on selected groups are listed in the table. Graphs of the results summarized in the table can be found in the right-side columns of supplemental Fig. 4, and the associated figure legends also list the statistical test used for each graph. The lower half of the figure shows the most relevant comparison in graphs: monocytes mono-cultured vs. monocytes co-cultured with Nskin/Kscar models. An ordinary one-way ANOVA with Tukey’s multiple comparisons was performed for CD14, CD11c, CD68, CD40/CD68 and CD206/CD68. The Kruskal–Wallist test with Dunn’s multiple comparison test was performed for CD1a. *p < 0.05, **p < 0.01, ****p < 0.0001. *Nskin* normal skin, *Kscar* keloid scar, *mo* monocytes, *momed* monocytes cultured in monocyte medium (for contents, see supplemental Table 1). *SE* skin equivalent comprising keratinocytes forming an epidermal layer on top of fibroblast-populated MatriDerm^®^; second batch of monocytes co-cultured with full skin equivalent from *t* = week 3 to week 5

It was first determined whether the medium used to culture the skin models influenced the monocyte phenotype. For this reason, monocytes were cultured in either a typical monocyte culture medium or different media used during skin model culture (FSM-I during dermal equivalent stage; KC-I/KC-II during the full skin equivalent stage; see also Fig. 1b and supplemental Table 1). There were no significant differences between monocytes cultured in our skin model medium (dermal or full skin equivalent stage) or the monocyte-specific medium (Fig. 4, supplemental Fig. 3 and 4). Notably, regardless of culture media used or whether monocytes were mono- or co-cultured, monocytes differentiated into a macrophage-like cells after 2 weeks of in vitro culturing: monocytes were initially CD11c^high^/CD14^high^/CD68^low^, but became CD11c^high^/CD14^low^/CD68^high^. Co-culture of monocytes with either normal skin or keloid scar models (at both the dermal and skin equivalent stage) did not further influence this phenotypic change (Fig. 4; supplemental Figs. 3 and 4), indicating that the transition was entirely due to the in vitro culturing process and was independent of the type of media used or co-culture conditions.

### Co-culture with reconstructed normal skin and keloid models skews monocyte differentiation towards M2 macrophage-like phenotype

Next we determined whether co-culture with skin models skewed monocytes to a macrophage type 1 (pro-inflammatory) or type 2 (tissue remodelling) phenotype. Co-culturing with either reconstructed normal skin or keloid skin equivalents generated macrophages that were predominantly CD68+/CD206+, which is characteristic of the more fibrotic M2-type macrophages (Fig. 4; supplemental Figs. 3 and 4). CD68+/CD40+M1-type macrophages made up only a small insignificant fraction of the total number of macrophages. The dermal equivalents induced a similar immunophenotype in the co-cultured monocytes as the full thickness reconstructed normal skin and keloid scar models. As can be seen in Fig. 4, the switch to the CD68+/CD206+M2 phenotype was only observed when monocytes were co-cultured with skin models (N SE + N mo, or Ks SE + Ks mo) and not when monocytes were cultured alone (N mo or Ks mo), the differentiation towards M2 was therefore not an in vitro culturing artefact.

It was also determined whether the monocytes had differentiated into fibroblasts (CD90+/fibronectin+), myofibroblasts (CD90/fibronectin/α-SMA), fibrocytes (classic staining: CD34+/collagen I+ and CD45+/collagen I+; new markers: MRP8+/PM2K−/CD45RO+/25F9+ stained intra- and extracellularly), and α-SMA positive fibrocytes (CD34+/α-SMA+/collagen I+ and CD45+/α-SMA+/collagen I+). Overall, there were negligible numbers of fibrocytes or fibroblasts present in all experimental monocyte conditions (supplemental Fig. 5a, b). To summarize, culturing monocytes in vitro induced a CD68+ macrophage-like phenotype, which further differentiated into a CD68+/CD206+M2 macrophage-like cell when co-cultured with either reconstructed normal skin or keloid models. Co-culture with dermal equivalents showed similar, but less pronounced results in monocyte phenotype (see supplemental Figs. 3 and 4).

## Discussion

In this study, we developed an immunocompetent keloid and normal skin model by reconstructing skin models in the presence of monocytes derived from the peripheral blood of individuals with and without a history of keloid scar formation, respectively. Notably, monocytes co-cultured with the reconstructed keloid scar as well as reconstructed normal skin skewed towards the M2 macrophage-like phenotype typically involved in tissue remodelling [8] and associated with fibrosis [24, 25], by becoming CD68+/CD206+ [34]. This indicates that monocytes within the vicinity of early regenerating/repairing tissues differentiate into M2 macrophages independent of whether the individual is prone to keloid or normotrophic scar formation.

The keloid in vitro models of our earlier studies [17, 18] identified a number of intrinsic abnormalities in keloid keratinocytes and fibroblasts, and showed differences compared to normal skin and normotrophic scar, but also compared to hypertrophic scar model. Compared to the normal skin model, the in vitro keloid scar model showed an increased contraction, dermal thickness (trend) and α-SMA staining, as well as decreased secretion of HGF and decreased dermal expression of the ECM-related genes *COL4A2*, *HAS1* and *MMP3*. Although the keloid scar model co-cultured with monocytes showed the same increased contraction and α–SMA expression with a trend towards increased dermal thickness, in this current study there was no further exaggeration of the keloid phenotype as we had hypothesized. Several immune cell types have been reported to be increased in keloid tissue, e.g. T-lymphocytes which were often concomitantly increased with the macrophages [3, 11, 31]. It is certainly possible that other immune cells besides monocytes/macrophages are required for effect influence on the keloid phenotype, likely even a combination of several immune cell types. Additionally, in contrast with the previously published basic keloid scar model (cultured without monocytes), we did not see decreased HGF secretion. The reason for this is unknown, possibly the smaller sample size in this study (*n* = 3) was insufficient to generate enough power to show statistically significant differences.

During wound healing, monocytes migrate from peripheral blood into the wound area where they differentiate into macrophages. Different macrophage phenotypes have been recognized, with the classically activated M1 and the alternatively activated M2 macrophages representing the two extremes of a continuum. M1 macrophages are usually active in the early stages of wound healing where they have pro-inflammatory, phagocytic and bactericidal effects. Conversely, M2 macrophages come to play in the tissue repair stage where they switch off the inflammatory response, regulate re-vascularization and, more importantly, regulate scar formation by stimulating fibroblast proliferation, myofibroblast differentiation and collagen deposition. As such, they play an important role in the final scar outcome [8, 34]. In this light, it should be noted that M2 macrophages have also been associated with fibrotic processes [24, 25]. Using CD40+ and CD206+ (also known as mannose receptor) as markers to identify M1 and M2 macrophages, respectively [34], we found that both normal skin and keloid scar models were able to induce the M2 phenotype in the co-cultured monocytes. It is important to note that this was not a culturing artefact, as mono-cultured monocytes in both the skin model medium and the monocyte medium only showed CD68 expression, but not CD206 expression. However, we had expected the keloid scar model to induce more M2 macrophage differentiation in comparison to the normal skin model. Others have reported an increase in M2 macrophages specifically in keloid scars. Using CD163+ as an M2 marker, Bagabir et al. [1] found that M2 macrophage staining was significantly upregulated in mature keloid scar tissue compared to normotrophic scars and normal skin. In another study, gene expression levels of IL-12 and iNOS or IL-10 and TGF-β were used to identify M1 and M2 macrophages, respectively [11], in macrophages isolated via enzymatic digestion of the keloid scar tissue (unspecified scar age). While they found the gene expression of all the aforementioned M1 and M2 markers to be increased, the M2-associated genes showed even higher levels of expression than the M1 markers. Although the presence of α-SMA positive myofibroblasts in keloids is still disputed [6, 13], we [article submitted] and others [12, 14, 28] have observed their presence in keloid scars. Considering that M2 macrophages also promote myofibroblast differentiation [8], this may have a cumulative effect further augmenting the development of excess scar tissue. Although M2 macrophages were also present in the co-cultures with normal skin models, there were only few myofibroblasts in the dermal compartment and therefore little to no myofibroblast target for the M2 macrophages to stimulate.

A possible reason for the observation that both reconstructed keloid scars as well as reconstructed normal skin were able to induce an M2 macrophage phenotype could be because co-culturing took place during the early reconstruction phase of the skin tissues. This means we are looking at early tissue formation here rather than the microenvironment of a maturing scar. If the keloid phenotype is sustained or exaggerated by M2 macrophages, this may only come into play at a later stage over the course of the 1–2 year duration of the remodelling phase [19], when the macrophages that would normally start to regress from the site of injury in a normal scar but may remain in keloid scars. Towards the end of culturing at 5 weeks, the skin models will have only just started to enter the remodelling phase, at which stage these differences may not yet have developed. Additionally, 1 × 10^6^ monocytes may have been an insufficient quantity to affect significant change in the keloid phenotype, while the significant diffusion distance may have also limited the effect of the skin models on monocyte differentiation. This may also be the reason for the minimal yield in fibrocytes, although Naylor et al. [23] offer another possibility. They suggest that fibrocytes may differentiate from a yet to be defined PBMC subtype and this may very well mean that the monocytes used in this study may not be the main source of fibrocyte precursors.

PBMCs and monocytes from patients with a history of keloid formation have previously been isolated and studied in co-culture systems. Liao et al. [15] used PBMC-derived CD14+ monocytes from keloid patients or healthy volunteers and co-cultured these with healthy dermal fibroblasts (2D culture) to study monocyte influence on fibroblast MCP-1 expression and proliferation rate. Both keloid PBMCs and CD14+ monocytes were shown to increase CCL2 (MCP-1) secretion more so than PBMCs and monocytes from normal scar-forming subjects. Via MCP-1 expression, the CD14+ monocytes were able to increase fibroblast proliferation and, more importantly, this effect was of a significantly larger magnitude when the CD14+ monocytes were derived from keloid patients. Only Jin et al. [11] used solely keloid patients’ derived cells in their co-culture system, but both cell types were immune cells. CD14+ macrophages derived from keloid tissue, which were co-cultured with CD3+ T-lymphocytes from peripheral blood of keloid patients, were potent stimulators of regulatory T cell differentiation by significantly upregulating Foxp3 expression compared to macrophages from normal skin. Finally, mast cells from a leukaemia cell line have been reported to stimulate collagen I expression when co-cultured with keloid fibroblasts [38]. However, none of the above-mentioned studies have co-cultured human keloid-derived monocytes or other immune cells with keloid-derived keratinocytes and/or fibroblasts. To our knowledge, this is the first immunocompetent, full thickness in vitro keloid scar model.

At present, it is not known which soluble mediators mediate the phenotypic switch to M2 macrophages when monocytes are co-cultured with skin models; to determine this would therefore be a logical follow-up experiment. Likely candidates include the known activation factors capable of inducing M2 differentiation, namely IL-4, IL-10, IL-13 and TGF-β [8, 34]. The interleukins are mostly secreted by immune cells of the innate and adaptive immune system [4, 20, 29], but only IL-10 is also secreted by keratinocytes. IL-10 and TGF- β would therefore be interesting candidates to investigate first. Other future experiments that might further improve our immunocompetent keloid scar model would include reducing the diffusion distance between the monocytes and the skin models, using the full PBMC fraction instead of only the monocytes, as well as increasing the quantity of monocytes for (co-) culture. Reducing diffusion distance between the immune cells and the skin models can be accomplished relatively easily by reducing the depth of the plate inserts, thereby lowering the transwells deeper into the underlying wells and reducing the quantity of medium needed and the diffusion distance. Additionally, increasing the quantity of co-cultured monocytes, incorporating the monocytes into the skin models to allow for direct cell-to-cell contact, and inclusion of other immune cell types (e.g. T-lymphocytes) or even combinations thereof are worthwhile considerations. In keeping with the latter suggestion, we could also consider incorporating monocytes into the microfluidic compartment of a skin-on-a-chip model to allow for infiltration into the skin tissue [2]. However, such an integrated model incorporating the monocytes into the skin models would not allow for analysis of the skin model phenotype and the monocytes separately as is possible with this co-culture model, and when using organ-on-chip technology the number of cells available for flow cytometric analysis would be a limiting factor. It is also important to note that with the exception of one keloid donor, the skin models did not come from the same donor as the co-cultured monocytes. Due to logistical issues and time constraints it was not possible in this study to donor match the rest of the keloid models to the monocytes, but it would certainly not be impossible for future research efforts. Obtaining normal skin from healthy volunteers in addition to obtaining significant volumes of blood for monocyte isolation, however, raises several ethical concerns. The allogeneic nature of the co-cultured monocytes may therefore also affect our results, although thus far the phenotype of our *n* = 1 donor-matched keloid monocytes does not appear to differ from the unmatched monocytes. All things considered, there are several ways to construct an immunocompetent keloid scar model, each with their own advantages and disadvantages. The co-culture of immune cells with our basic keloid scar model as presented in this paper serves as a simple, yet elegant method to evaluate the individual and cumulative effects of additional cell types on keloid scar formation.

## Electronic supplementary material

Below is the link to the electronic supplementary material.

Supplementary material 1 (DOCX 4351 kb)

Supplementary material 2 (DOCX 19 kb)

## Abbreviations

α–SMA: Alpha smooth muscle actin
DE: Dermal equivalent (3-week-old skin model, comprising only the dermal compartment)
Ks/Kscar: Keloid scar
momed: Monocyte medium
N/Nskin: Normal skin
PBMCs: Peripheral blood mononuclear cells
SE: Skin equivalent (5-week skin model, comprising the epidermal and dermal compartment)
SEM: Standard error of the mean
