# Supplementary material for: Monocytes co-cultured with reconstructed keloid and normal skin models skew towards M2 macrophage phenotype
Source: Arch Dermatol Res. 2019 Jun 11;311(8):615–27. doi: 10.1007/s00403-019-01942-9 (PMC6736899; doi:10.1007/s00403-019-01942-9)

**Supplemental figure 1. Polycarbonate plate insert placed on top of a 6-well plate.** These plate inserts were designed to elevate the transwells (diameter 24 mm), thereby increasing the distance between the transwell membrane and the underlying well. This in turn, increases the volume of medium for air-exposed culture from 2 ml to 10 ml, which was required for co-culture of the skin equivalents with monocytes. Upper right photo shows the plate insert alone, the lower right photo shows the plate insert when used with the transwells and the 6-wells plate. Left photo zooms in on the transwells placed on top of the plate insert.


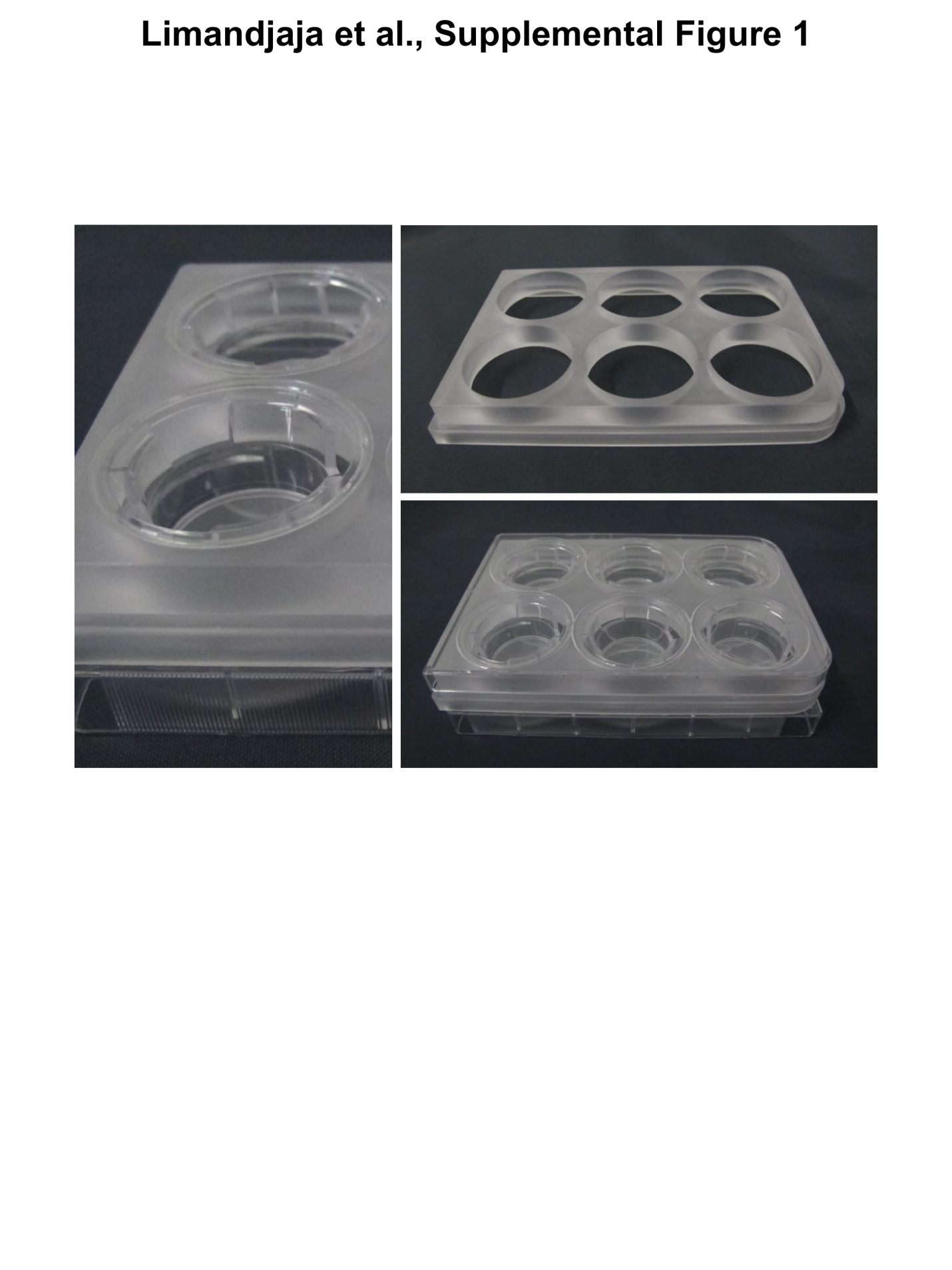


**Supplemental figure 2. Contraction, epidermal thickness, dermal thickness.** Results of contraction, epidermal and dermal thickness from the table in fig. 2 displayed as graphs. Results are presented as mean ± SEM for n=3 normal skin (Nskin) and n=3 keloid scars (Kscar) skin equivalents (SE), cultured with or without monocytes (mo). (A) shows contraction measured as a reduction in absolute surface area after 5 weeks of culturing; (B) shows the dermal thickness measured in μm; (C) shows the number of viable epidermal cell layers in the SE. An ordinary one-way ANOVA with post-hoc Tukey’s multiple comparisons test was performed, *p < 0.05.


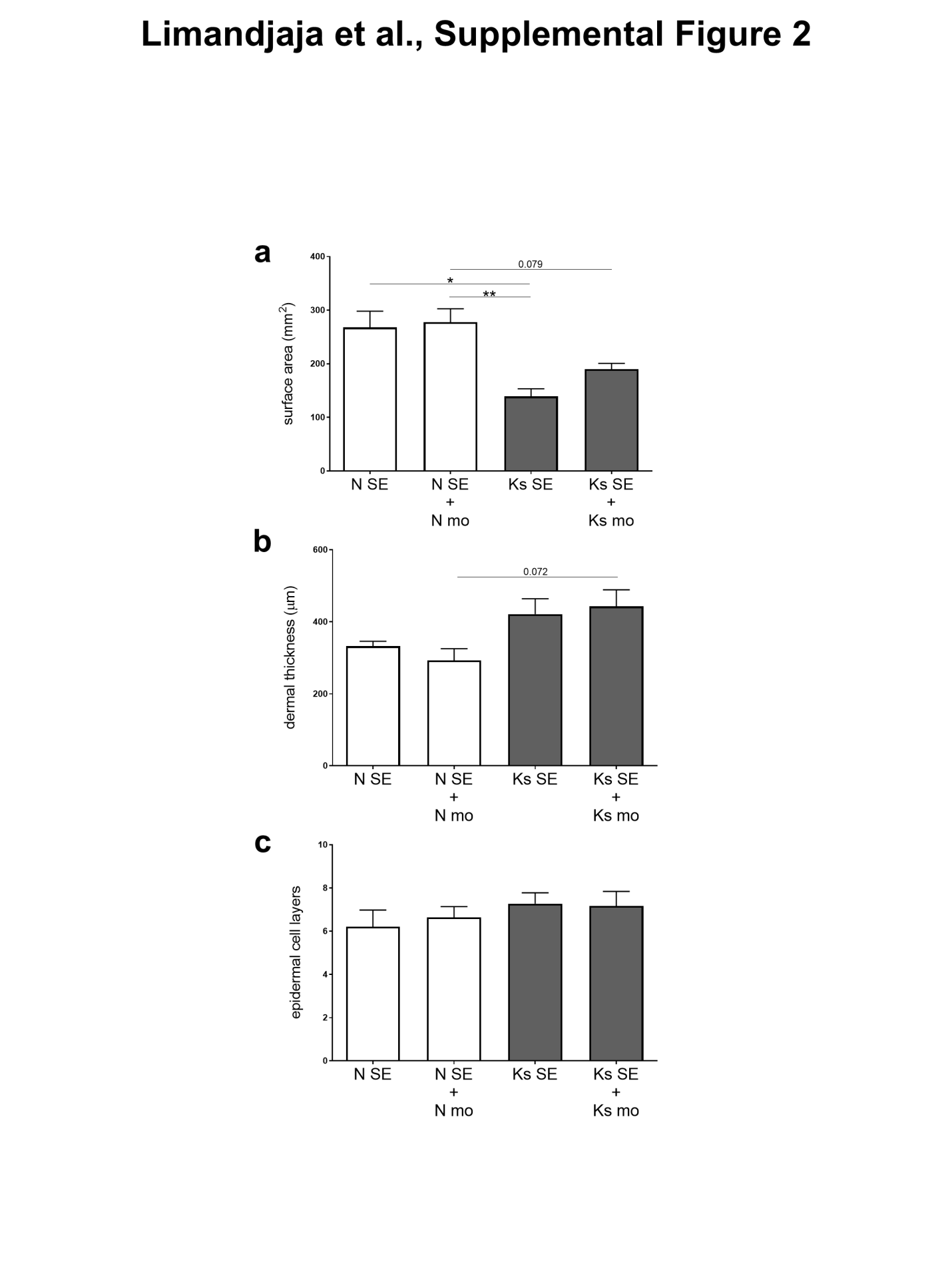


**Supplemental figure 3. Immunophenotyping of monocytes (co-) cultured with dermal equivalents: monocyte, dendritic cell and macrophage marker expression.** Monocytes, both mono- and co-cultured with dermal equivalents, were analysed for monocyte (CD14+, CD11c+), dendritic cell (CD1a+), macrophage (CD68+), M1 macrophage (CD40+) and M2 macrophage (CD206+) marker expression via FACS analysis. The table summarizes the results of each experimental group by listing the mean % positive staining ± SEM with n=3 for all 4 normal skin conditions and n=3 for all 4 keloid scar experimental groups except when indicated by **†**: n=2 donors; vs.: versus (compared to).. Statistically significant results of an ordinary one-way ANOVA or Kruskal-Wallist test with post-hoc testing on selected groups, are listed in the table. Graphs of the results summarized in the table can be found in the left-side columns of supplemental figure 4, the associated figure legends also list the statistical test used for each graph. The lower half of the figure shows the most relevant comparison in graphs: monocytes mono-cultured vs. monocytes co-cultured with Nskin/Kscar models. An ordinary one-way ANOVA with Tukey’s multiple comparisons test was performed for CD14, CD11c, CD1a, CD68, CD40/CD68 and CD206/CD68. *p < 0.05, **p < 0.01, ****p < 0.0001. Nskin: normal skin, Kscar: keloid scar, mo: monocytes, momed: monocytes cultured in monocyte-medium (for contents, see supplementary table 1). DE: dermal equivalent comprising fibroblasts in MatriDerm®, monocytes co-cultured with dermal equivalent from t = week 1 – week 3.


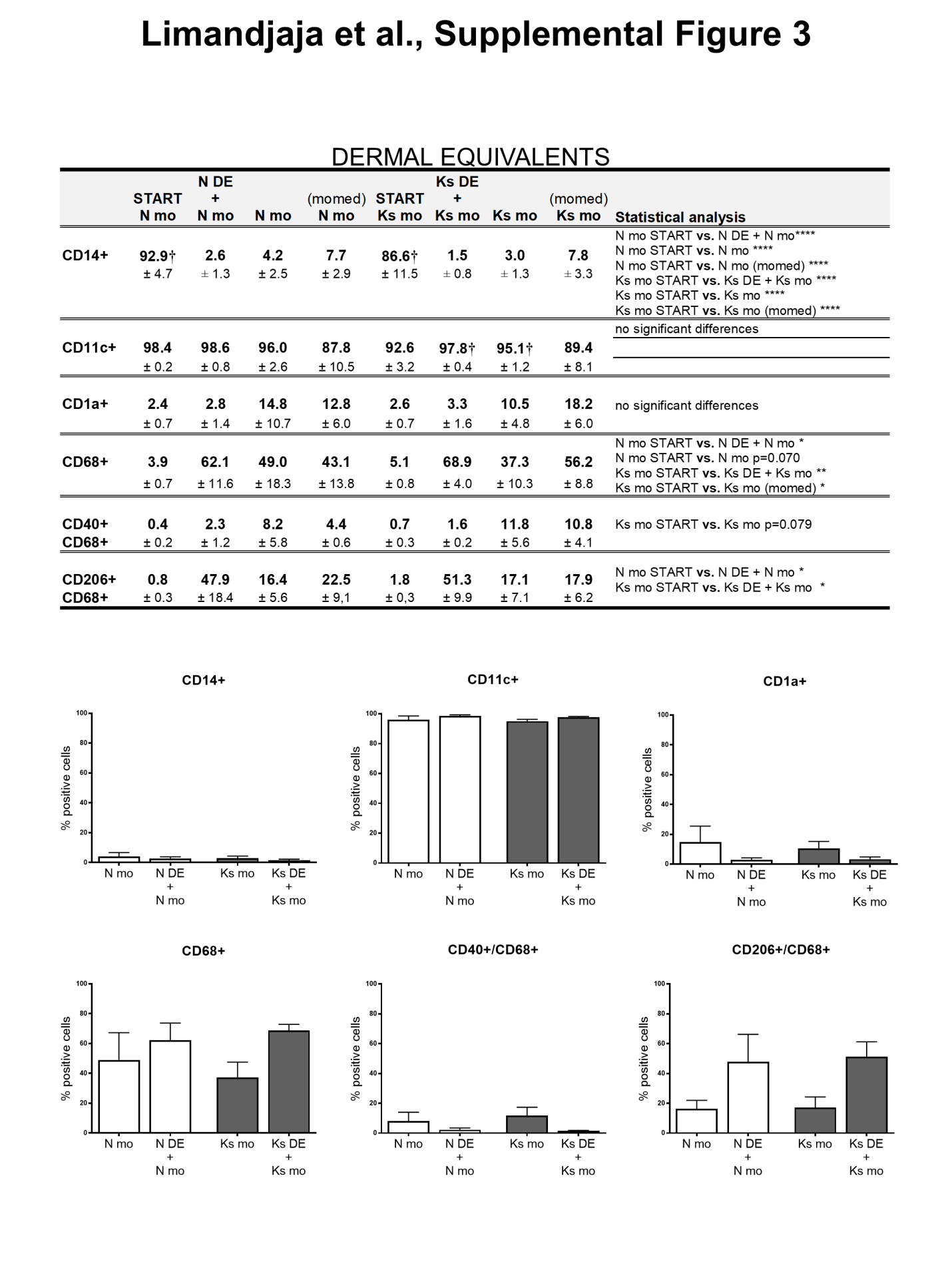


**Supplemental figure 4. Immunophenotyping of (co-) cultured monocytes: monocyte, dendritic cell and macrophage marker expression.** Graphs of results summarized in the tables of fig. 4 (right-side columns) and supplemental fig. 3 (left-side columns). Mono- or co-cultured monocytes were stained for (A) CD14, CD11c, CD1a and (B) CD68, CD40/CD68, CD206/CD68. Results were presented as the mean % positive staining ± SEM, with n=3 donors for each experimental condition except when indicated on the x-axis of the graphs by **†**: n=2 donors. An ordinary one-way ANOVA with Tukey’s multiple comparisons test on selected pairs (dermal equivalents: CD14+, CD1a+, CD68+, CD206+/CD68+; skin equivalents: CD1a+, CD68+, CD40+/CD68+) or the Kruskal-Wallis test with Dunn’s multiple comparisons test on selected pairs (dermal equivalents: CD11c+, CD40+/CD68+; skin equivalents: CD14+, CD11c+, CD206+/CD68+). *p < 0.05, **p < 0.01, ****p < 0.0001. Nskin: normal skin, Kscar: keloid scar, mo: monocytes, momed: monocytes cultured in monocyte-medium (for contents, see supplementary table 1). DE: dermal equivalent comprising fibroblasts in MatriDerm®, monocytes co-cultured with dermal equivalent from t = week 1 – week 3. SE: skin equivalent comprising keratinocytes forming an epidermal layer on top of fibroblast-populated MatriDerm®, second batch of monocytes co-cultured with full skin equivalent from t = week 3 – week 5.


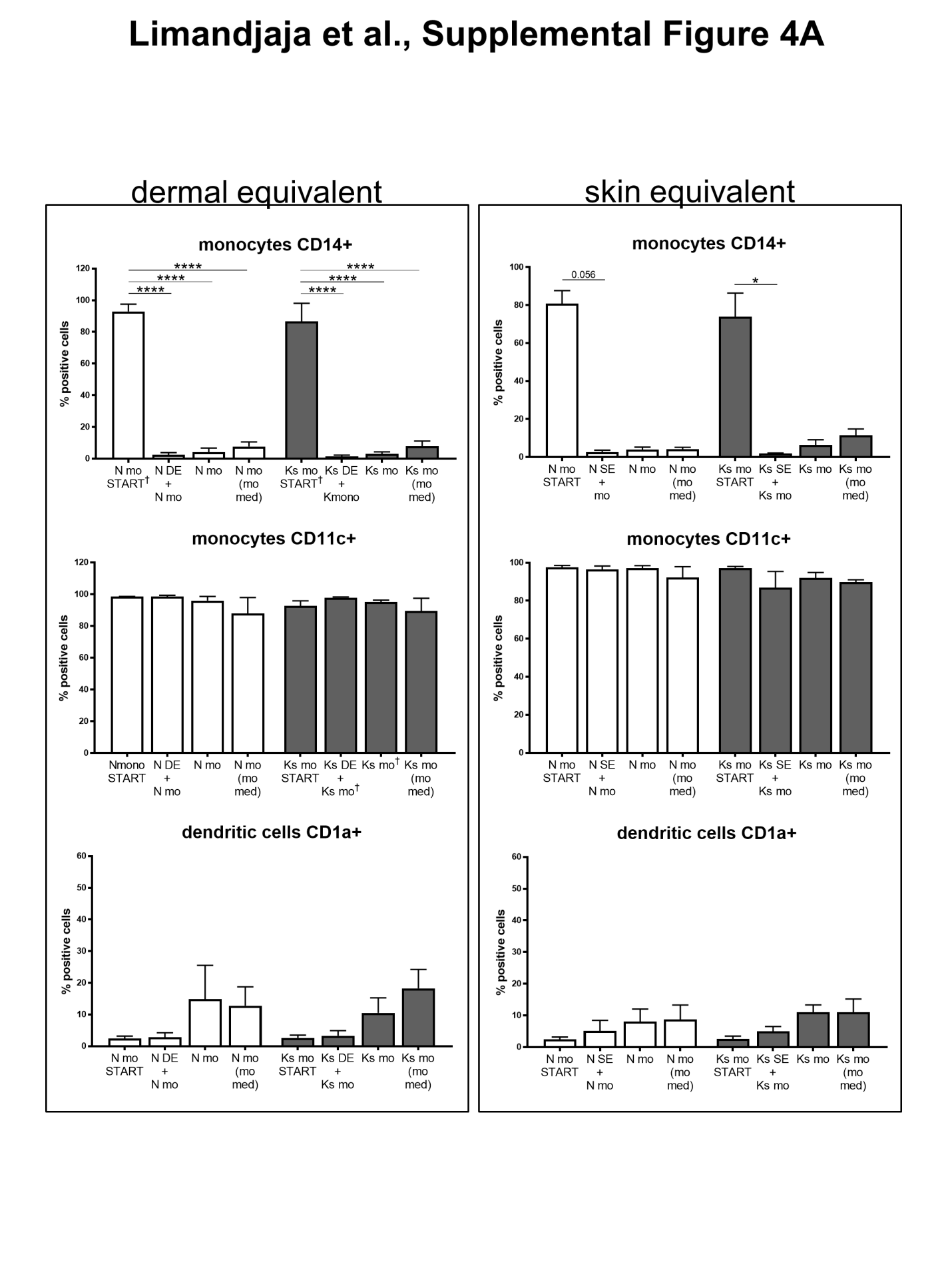

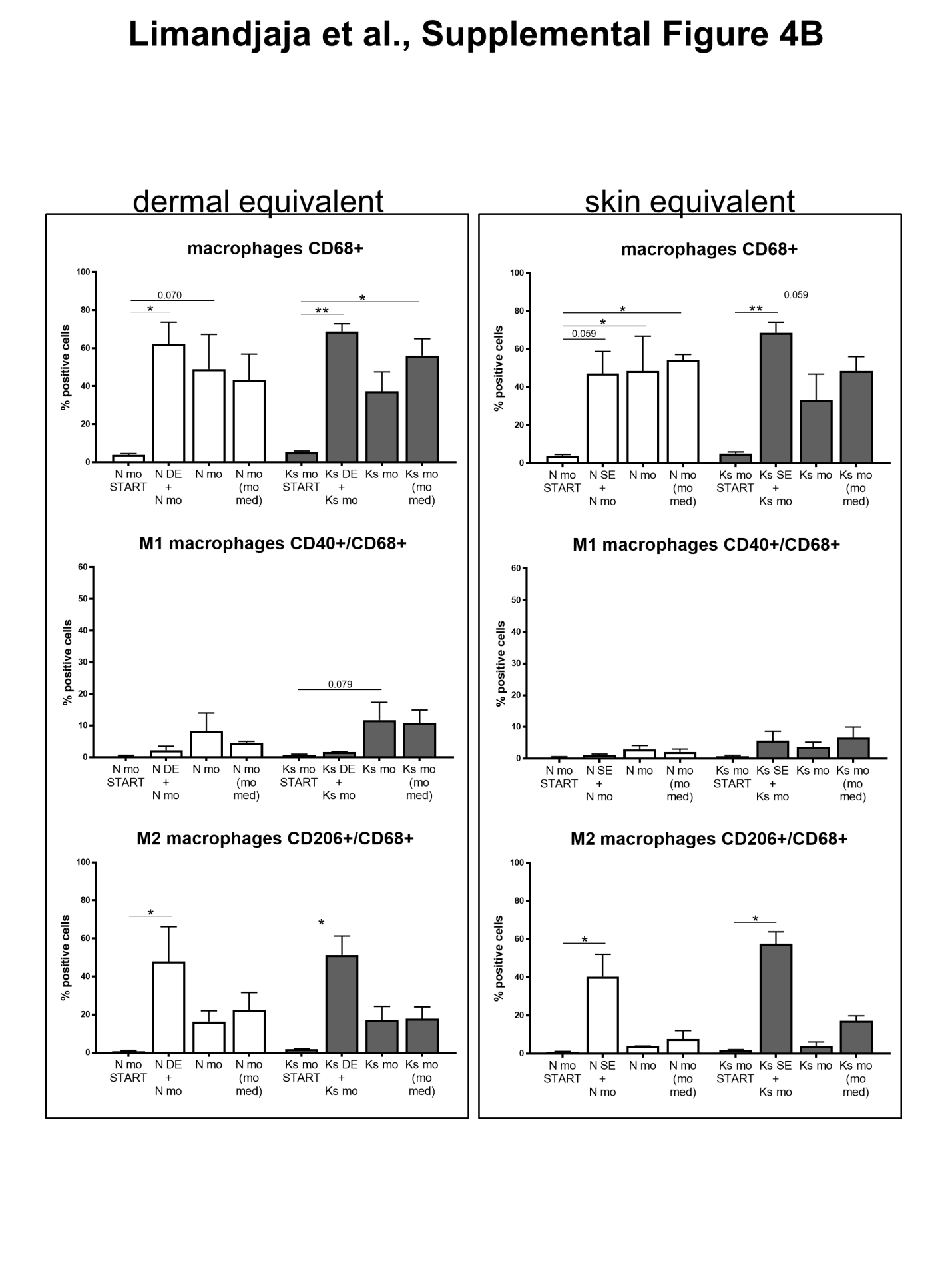


**Supplemental figure 5. Immunophenotyping of (co-) cultured monocytes: fibrocyte and fibroblast marker expression.** Mono- or co-cultured monocytes were stained for (A) fibrocyte (CD34+/LSP1+/collagen1+, CD45+/LSP1+/collagen1+, MRP8/14+/PM2K−/CD45RO+/25F9+) and fibroblast (CD90+/fibronectin+) markers, (B) α-SMA positive fibrocytes (CD34+/LSP1+/collagen1+, CD45+/LSP1+/collagen1+) and myofibroblast (α-SMA+/CD90+/fibronectin+) markers. Results were presented as the mean % positive staining ± SEM, with n=3 donors for each experimental condition except when indicated on the x-axis of the graphs by **†**: n=2 donors. An ordinary one-way ANOVA with Tukey’s multiple comparisons test on selected pairs (dermal equivalents: CD45+/LSP1+/collagen1+; skin equivalents CD45+/LSP1+/collagen1+, α-SMA+/CD34+/LSP1+/collagen1+, α-SMA+/CD45+/LSP1+/collagen1+, MRP8/14+/PM2K−/CD45RO+/25F9+) or the Kruskal-Wallis test with Dunn’s multiple comparisons test on selected pairs (remainder of graphs not previously listed). Nskin: normal skin, Kscar: keloid scar, mo: monocytes, momed: monocytes cultured in monocyte-medium (for contents, see supplementary table 1). DE: dermal equivalent comprising fibroblasts in MatriDerm®, monocytes co-cultured with dermal equivalent from t= week 1 – week 3. SE: skin equivalent comprising keratinocytes forming an epidermal layer on top of fibroblast-populated MatriDerm®, second batch of monocytes co-cultured with full skin equivalent from t= week 3 – week 5.


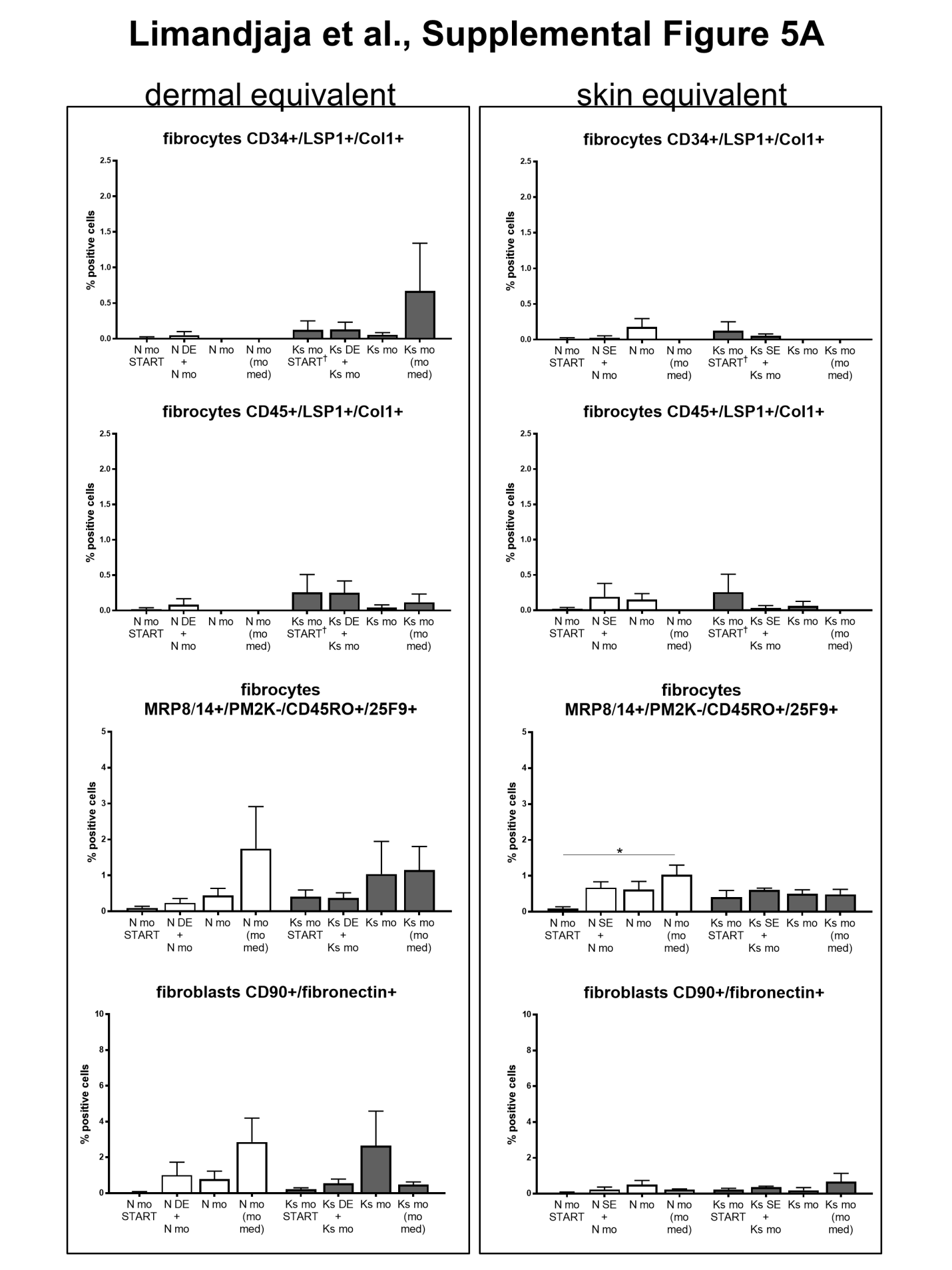


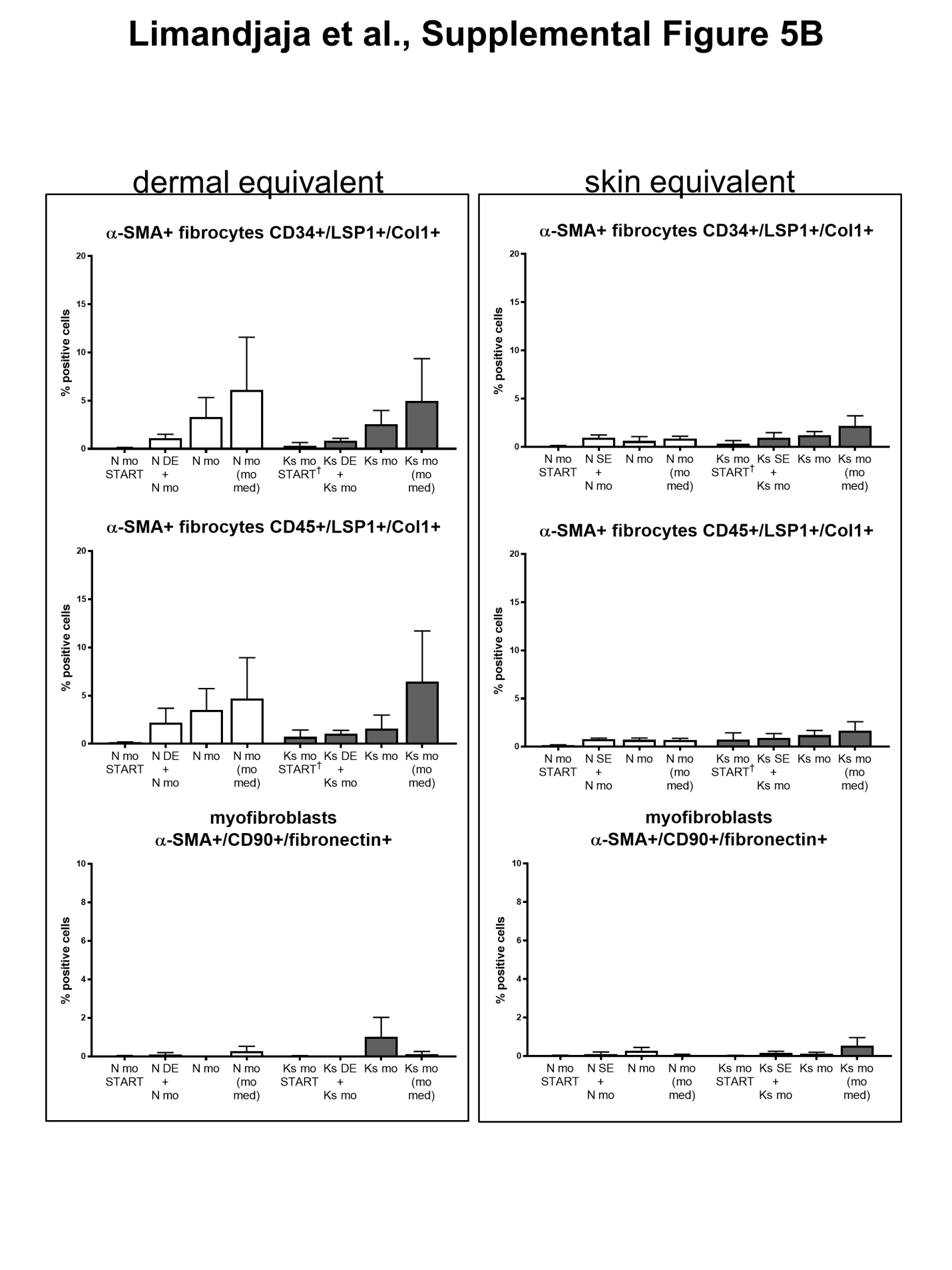

Supplement: Supplementary file 1 — Supplementary material 1 (DOCX 4351 kb) [file 403_2019_1942_MOESM1_ESM.docx]
