# Supplementary material for: Monocytes co-cultured with reconstructed keloid and normal skin models skew towards M2 macrophage phenotype
Source: Arch Dermatol Res. 2019 Jun 11;311(8):615–27. doi: 10.1007/s00403-019-01942-9 (PMC6736899; doi:10.1007/s00403-019-01942-9)
Supplement: Supplementary file 2 — Supplementary material 2 (DOCX 19 kb) [file 403_2019_1942_MOESM2_ESM.docx]

**Supplemental table 1**: culture media

| **Medium** | **Components** |
| --- | --- |
| KC-I | DMEM (Lonza, Verviers, Belgium) : F12-HAM nutrient mixture + L-glut. (HAMF12; Gibco, Grand Island, NY, USA) in a 3:1 ratio with 1% UltroserG (Biosepra, Cergy-St-Christophe, France), 1% PenStrep (Gibco, Grand Island, USA), 2ng/ml human Keratinocyte Growth Factor, 0.09 µmol/L Insulin, 1µmol/L Hydrocortisone, 1µmol/L Isoproterenol |
| Fibroblast medium | DMEM (Lonza), 1% UltroserG (Biosepra), 1% PenStrep (Gibco) |
| FSM-I | DMEM (Lonza), 2% UltroserG (Biosepra), 1% PenStrep (Gibco), 5µg/ml Insulin 10-3M, 50µg/ml L-Ascorbic acid and 5ng/ml Epidermal Growth Factor |
| FSM-II | DMEM:HAMF12 (Lonza; Gibco) in a 3:1 ratio, 1% PenStrep (Gibco), 2% UltroserG (Biosepra), 5 µg/ml Insulin 10-3M, 50µg/ml L-Ascorbic acid and 5ng/ml Epidermal Growth Factor |
| KC-II | DMEM:HAMF12 Lonza; Gibco) in a 3:1 ratio, 1% PenStrep (Gibco), 0.2% UltroserG (Biosepra), 0.1 µmol/L Insulin, 1µmol/L Hydrocortisone, 1µmol/L Isoproterenol hydrochloride, 10 µmol/L L-Carnitine hydrochloride, 0.01 µmol/L L-Serine, 1µmol/L DL-α-Tocopherol, 0.4mmol/L L-Ascorbic acid, supplemented with a lipid mixture containing 7 µmol/L Arachidonic acid, 25 µmol/L Palmitic acid, 15 µmol/L Linoleic acid and 24 µmol/L Bovine Serum Albumin |
| Monocyte medium | DMEM (Lonza), 10% heat inactivated FBS (HyClone®, Northumberland, UK), 1% PenStrep (Gibco) |
| Medium for collection of 24-hr supernatant  after 5 weeks | Skin equivalents with/without monocytes, monocytes cultured in KC-II: KC-II without Hydrocortisone  For monocyte monocultures cultured with monocyte medium: monocyte medium |

**Supplemental Table 1**: shows an overview of all the different culture media used for the construction of the skin models and monocytes co-cultures. All reagents were obtained from Sigma-Aldrich (St. Louis, MO, USA) unless otherwise specified. Whilst in monoculture, keratinocytes and fibroblasts were cultured in KC-I and fibroblast medium respectively. The skin models with or without monocytes were cultured in FSM-I (for fibroblast seeding into MatriDerm®), FSM-II (t = 0-3 weeks after seeding), KC-I (for 3 days after seeding of keratinocytes onto the fibroblast-populated MatriDerm®) and KC-II (from air-exposure until end of culture) subsequently; see timeline in fig. 2A. Mono-cultured monocytes were also cultured in the same medium as the skin models: with the first batch of monocytes (cultured during week 1–3) cultured in FSMI-II and the second batch (cultured during week 3-5) cultured briefly (3 days) in KC-I, but mostly in KC-II medium. As an additional control group, monocytes were also cultured in their own monocyte medium.

**Supplemental table 2**: Flow cytometry antibodies

| **FL-1** | **FL-2** | **FL-3** | **FL-4** |
| --- | --- | --- | --- |
| CD1a (Fitc)  Mouse monoclonal IgG1  555806  BD BIosciences | Langerin (PE)  Mouse monoclonal IgG1  PN IM3577  Beckman Coulter | CD14 (PerCp)  Mouse monoclonal IgG2b  345786  BD BIosciences | CD11c (APC)  Mouse monoclonal IgG2b  333144  BD Biosciences |
| CD40 (Fitc)  Mouse monoclonal IgG1  555588  BD BIosciences | CD11c (PE)  Mouse monoclonal IgG1  12-0116-41  eBioscience | CD206 (PerCp-Cy5.5)  Mouse monoclonal IgG1  321122  BioLegend | 25F9 (effluor660)*  Mouse monoclonal IgG1  50-0115-41  eBioscience |
| CD34 (Fitc)  Mouse monoclonal IgG1  555821  BD BIosciences | CD68 (PE)  Mouse monoclonal IgG2b  556078  BD BIosciences | LSP-1 (PE-Cy7)**  Rabbit polyclonal IgG  bs-5154R-PE-Cy7  Bioss, | Collagen I (APC)**  Mouse monoclonal IgG1  NBP1-05171  Novus Biologicals  +  Goat anti-Mouse IgG APC  731857  Beckman Coulter |
| CD45 (Fitc) Mouse monoclonal IgG1  555482  BD BIosciences | α-SMA (PE)**  Mouse monoclonal IgG2a  IC1420P  R&D systems | CD45RO (PE-Cy5)*  Mouse monoclonal IgG2a  555494  BD Biosciences | CD56 (APC)  Mouse monoclonal IgG2a  Ab28335  Abcam |
| MRP8/14 (Fitc)*  Mouse monoclonal IgG1  BM4025I  Acris antibodies | ***PM-2K****  *Mouse monoclonal IgG1*  *Ab58822*  ***+***  ***Goat anti-Mouse IgG1 (PE)***  *Ab99912*  *Abcam* | CD90 (PerCp-Cy5.5)  Mouse monoclonal IgG1  45-0909-41  eBioscience |  |
| Fibronectin (AF488)**  Mouse monoclonal IgG1  563100  BD Biosciences | CD19 (PE)  Mouse monoclonal IgG1  561741  BD Biosciences | CD123 (PerCp-Cy5.5)  Mouse monoclonal IgG1  306016  BioLegend |  |
| CD3 (Fitc)  M1466 Pelicluster  Sanquin |  |  |  |

**Supplemental table 2:** Antibodies used for the immunophenotyping of the co-cultured monocytes by way of flow cytometry, followed by clonality, catalogue number and manufacturer. Italicized text: two-step staining procedure; * stained intracellularly and extracellularly, ** always stained intracellularly. Corresponding isotype antibodies were used as a control for each of the listed antibodies.
